# Observational Study of Pediatric Inpatient Pain, Nausea/Vomiting and Anxiety

**DOI:** 10.3390/children6050065

**Published:** 2019-05-03

**Authors:** Michael Schlegelmilch, Salima Punja, Hsing Jou, Andrew S. Mackie, Jennifer Conway, Bev Wilson, Maria Spavor, Dawn Hartfield, Sunita Vohra

**Affiliations:** 1 Faculty of Medicine and Dentistry, University of Alberta, Edmonton, AB T6G 2R3, Canada; mschlege@ualberta.ca (M.S.); hjou@ualberta.ca (H.J.); mackie1@ualberta.ca (A.S.M.); conway@ualberta.ca (J.C.); Bev.Wilson@albertahealthservices.ca (B.W.); mspavor@ualberta.ca (M.S.); dawn.hartfield@albertahealthservices.ca (D.H.); 2 Integrative Health Institute, University of Alberta, Edmonton, AB T6G 2C8, Canada; punja@ualberta.ca

**Keywords:** pediatric, hospital, pain, nausea, vomiting, anxiety

## Abstract

Background: The prevalence and severity of pain, nausea/vomiting, and anxiety (PNVA) among hospitalized children is not well established. We describe the prevalence and severity of PNVA among hospitalized patients from oncology, general pediatrics, and cardiology services in a tertiary care center. Methods: Patients were recruited on admission and enrolled if their caregiver consented, spoke English, and were anticipated to stay 2–30 days. Symptoms were measured weekdays using age-validated tools. PNVA symptoms were described and compared. Results: We enrolled 496 (49.4%) patients of 1005 admitted. Patients were predominantly Caucasian (57.9%) on their first admission (53.6%). The average (SD) age was 8.6 years (5.9) in oncology, 4.2 (5.3) in general pediatrics and 2.6 (4.0) in cardiology. 325 (65.6%) patients reported anxiety, 275 (55.4%) reported nausea and 256 (52.0%) reported pain. Mean (SD) severity out of 10 was 3.7 (2.5) for anxiety, 3.2 (2.1) for nausea and 3.0 (1.5) for pain. Prevalence of PNVA was no different between clinical programs, but pain (*p* = 0.008) and nausea (*p* = 0.006) severity were. PNVA symptom co-occurrence was positively correlated (*p* < 0.001). Conclusions: Anxiety was the most common and severe symptom for hospitalized children. Patients in oncology demonstrated the least severe pain and nausea with no difference in anxiety between services.

## 1. Introduction

Pediatric disease-specific symptom prevalence and severity studies have been widely published, such as pain in musculoskeletal conditions, nausea in functional gastrointestinal disorders, and anxiety in type one diabetes mellitus [1,2,3]. Negative effects of untreated pain can be physiologic, psychological, and emotional. Unfortunately, pain in children is under assessed, under treated and costly [4,5]. Researchers have stated a need for more systematic and dedicated pain assessment and management for hospitalized children [6,7]. Recent literature has demonstrated sustained improvements in pediatric inpatient pain management practices following dedicated quality improvement and knowledge translation initiatives [8]. Similarly, nausea is well researched in specific settings such as post-operative recovery rooms and in patients receiving emetogenic medications, often in oncology [9,10]. Anxiety, though, is less well examined, especially in hospitalized patients who were not specifically undergoing a painful procedure [11].

Management for pain, nausea/vomiting and anxiety (PNVA) likewise has been well researched for specific instances of PNVA in specific illnesses. However, our search of the literature did not yield any manuscripts that describe PNVA prevalence and severity for hospitalized pediatric inpatients. Therefore, we sought to describe pediatric PNVA prevalence and severity among patients admitted to three participating hospital inpatient services, in the hopes that a better understanding of PNVA prevalence and severity will result in improved treatment. Under-recognition of pain is a known barrier to adequate management [12]. This may apply to nausea and anxiety as well. Symptoms which are not recognized cannot be treated and may result in more intense subsequent symptom experiences, higher symptom rates post discharge, and greater health system costs [5,13,14]. Stemming from a desire to improve inpatient symptom management of pain, nausea/vomiting and anxiety (PNVA) we sought to establish the rates of these symptoms for inpatients at a large urban pediatric tertiary care hospital.

## 2. Materials and Methods

Our study is a single-center, cross-sectional observational study from February 2013 to January 2016, of patients admitted to oncology, cardiology or general pediatrics and treated with standard hospital practices at the Stollery Children’s Hospital in Edmonton, Canada. The Stollery is a quaternary care children’s hospital that provides a full range of cardiology, cardiac surgery services and oncology services with the exception of bone marrow transplant.

Children and caregivers were screened at admission to oncology, general pediatrics or cardiology, which included postoperative cardiac surgery patients. There were no other surgical programs in our study. Patients were included if the caregiver could communicate in English, was available to participate and was willing to provide informed written consent. Assent was obtained from the child, whenever possible. Participants were eligible for inclusion for both elective and non-elective admissions and included if their length of stay was predicted to be between 2 and 30 days and if their age at baseline was less than 17 years, as our symptom measurement tools were validated for patients up to 17 years of age. If a patient was discharged and re-admitted during the study period they were included as a new study participant.

Demographic and admission data were extracted from the patient charts retrospectively. A trained research nurse from our study team collected symptom data at baseline and daily until they were discharged, transferred, deceased, or 30 days had elapsed, whichever was soonest. All patient symptom score data were collected daily, on weekdays only. Since we were assessing multiple symptoms, each one was measured separately using age appropriate, validated tools. Patient pain was measured in pre-verbal children by parents using the behavioral observational Faces, Legs, Activity, Cry, Consolability (FLACC) [15] tool. Verbal children reported their own pain with the Faces Pain Scale—Revised (FPS-R) [16].

Verbal patients or parent proxies were asked to describe their pain, which was then categorized into types from a predetermined list (acute, sub-acute, chronic, procedural, post-operative, or other). Nausea and anxiety scores were collected from verbal children only using the Baxter Retching Faces [17] (BARF) and Pediatrics Anxiety Faces Scale [18,19] (PAFS), respectively. Pictorial faces tools have been found to be advantageous in the assessment of pediatric pain; they may also promote the assessment of other subjective symptoms, such as nausea and anxiety [18]. All tools compute an aggregate score out of 10.

Descriptive statistics were reported as numbers and percentages for categorical variables or means and standard deviations for continuous variables. Pain scores (FLACC and FPS-R) were analyzed together as continuous variables that ranged from 0–10, lower scores representing less severe symptoms. Daily PNVA symptom scores were collected each day and averaged to compute a mean score for that admission, both with and without zero (symptom free day) scores. Average symptom severity and prevalence were compared between groups with ANOVA for parametric data and Kruskall–Wallis for non-parametric. 

Daily medication administration was recorded and medications for PNVA were grouped into four: (1) Non-opioid analgesics: acetaminophen, acetylsalicylic acid, ibuprofen, indomethacin, ketorolac, and naproxen; (2) Opioids: hydromorphone, morphine, and fentanyl; (3) Anti-emetics: aprepitant, dimenhydrinate, granisetron, metoclopramide, nabilone, and ondansetron; and (4) Anxiolytics/sedatives were clonazepam, diazepam, lorazepam, midazolam, and melatonin. 

Study data were collected and managed using Research Electronic Data Capture (REDCap)^TM^ hosted at the University of Alberta [20]. 

Ethics approval was obtained from the University of Alberta Research Ethics Board (project identification code: Pro00010904; date of approval: 22 February 2011).

## 3. Results

### 3.1. Characteristics of Participants

**Figure 1:**
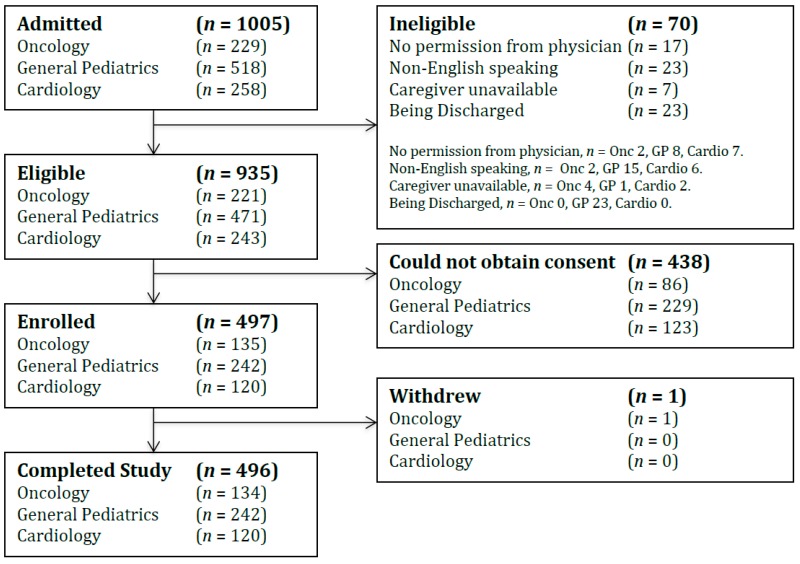
CONSORT patient enrolment flow diagram.

There were 1005 patients admitted to the three participating study services during the observation period. Seventy patients (7.0%) were ineligible due to lack of permission from the care team, lack of ability to communicate in English, or their length of stay was outside of the two- to 30-day range (Figure 1). Of the 935 eligible patients, 438 (46.8%) were not enrolled due to lack of assent/consent from the patient or caregiver. An observational cohort of 497 (53.2%) patients was enrolled, and 496 (99.8%) completed the study (Figure 1). Study participants were 48.2% female, 57.9% Caucasian, 8.7% First Nations, 8.5% Filipino with the remainder split between African, Arab, Chinese, East Indian, Latin American and Others. Participants had a mean (SD) age of 5.1 (5.7) years. The mean (SD; range) ages of patients in oncology, general pediatrics and cardiology were 8.6 (5.9; 18 days–17.5 years), 4.2 (5.3; 6 days–16.9 years) and 2.6 (4.0; 4 days–16.9 years) years respectively. The average non-verbal age was 1.3 years (range: 4 days to 15 years). The average verbal age was 11.5 years (range: 3.7 years to 17.5 years). The proportion of patients on their first admission was greatest in cardiology (57.5%), compared to general pediatrics (35.9%) and oncology (14.2%). The median (IQR) length of stay was 3 days (2–6) for oncology, 4 (3–8) for general pediatrics and 3 (2–9) for cardiology (Table 1). 

### 3.2. PNVA Prevalence, Severity and Medications

In total, 256 (52.0%) of the 496 participants reported pain at least once during their admission, 275 (55.4%) reported nausea and 325 (65.6%) reported anxiety. The mean (SD) percentage of days with anxiety was 46.0% (37.4), followed by nausea 32.3% (37.8) and pain 28.2% (35.8). When symptomatic, the mean (SD) symptom severity out of 10 was 3.7 (2.5) for anxiety, 3.2 (2.1) for nausea and 3.0 (1.5) for pain (Table 2).

Symptom prevalence was not significantly different between admitting services; however, the severity of pain and nausea was significantly different between services. Oncology patients had less severe pain (*p* = 0.008) and nausea (*p* = 0.006) compared to patients in general pediatrics and cardiology. However, there was no statistically significant difference between services for severity of anxiety (*p* = 0.11) (Table 3).

Medication rates of use and percentage of patient days with symptoms are displayed in Table 4. There was a significant difference between clinical programs in medication use for all medication types.

While many (32.8%) patients reported acute pain, the majority (48.4%) reported “other” type of pain, of which 27% did not further specify the nature of pain. Of those who did, abdominal pain (19.1%) (often related to being “nil per os”) was the most common source followed by generalized pain or irritability (14.3%) and intravenous catheter site pain (5.8%).

### 3.3. Predictors of PNVA Severity

Pain was more severe in patients who were rated their own symptoms (*n* = 185), compared to those who had proxy ratings (*n* = 311). The mean (SD) self-assessed FPS-R score was 1.3 (1.7) compared to 0.81 (1.4) for those rated by proxy (*p* < 0.001). Pain-nausea, pain-anxiety and nausea-anxiety correlations were statistically significant (*p* < 0.001); increased severity of any one symptom was correlated with a more severe co-occurring symptom of the PNVA cluster.

## 4. Discussion

It is well borne out in the literature that pain is an under-recognized and undertreated symptom in pediatric populations [13,14,21]. A systematic review by King et al. found that prevalence can range broadly from 4% to 83% [22]. Untreated or dismissed pain in childhood can have lasting impacts [18], including an increased risk of chronic pain that persists into adulthood [23,24]. Although nausea is reported less commonly than pain, the effect of nausea is well studied in some pediatric populations and is known to cause greater global distress [25]. Studies have examined acute anxiety, as it can be difficult to differentiate from pain in young children [26]; however, there are few dedicated studies examining the prevalence and severity of nausea and anxiety in broader pediatric patient populations. To the best of our understanding, we are the first to prospectively describe the prevalence and severity of PNVA in hospitalized children.

Interactions between symptoms are well researched, and our study corroborated what is known about symptom correlations. Correlations between changes in anxiety and pain are seen across many patient populations in acute and chronic settings. Studies have found that anxiety severity positively correlates with pain severity or pain related functioning, both in procedural pain and chronic pain populations [27,28,29,30,31,32,33,34]. Pharmacological and non-pharmacological management of anxiety prior to a medical procedure gives improved pain distress from procedural pain as well [35,36]. Not surprisingly, the interaction of anxiety and pain is not uni-directional, and anxiety can increase pain sensation, which in turn increases anxiety [34]. Considering that anxiety and pain also affect quality of life, concurrent treatment of both symptoms is recommended [37,38]. It is most surprising that we found anxiety was the most prevalent, severe and durable symptom for pediatric inpatients.

We found a difference between admitting services in the severity of pain and nausea but not anxiety, with oncology patients having the least severe symptoms. One possible reason for this is illness experience. Dupuis et al. (2016) showed that pediatric cancer pain and anxiety improved over a treatment course [39]. Another could be due to routine treatment protocols for childhood cancer. In North America, the Children’s Oncology Group (COG) makes specific recommendations for the assessment and management of common distressing symptoms, such as PNVA, for all children receiving chemotherapy. Interventions combine pharmacologic and adjunctive non-pharmacologic approaches such as acupuncture, guided imagery, progressive muscle relaxation, animal assisted therapy and others [39]. It is possible that a similar systematic approach to PNVA on general pediatric and cardiology units may be beneficial, although this has not been previously studied.

Our observational cross-sectional study indicates that PNVA symptoms are common in pediatric inpatient populations. An observational study of inpatient pain in adults suggested a comparatively higher pain prevalence (70.4%), yet similar severity (3.76) [40]. The preponderance of anxiety across the three patient groups may be attributable to the many aspects of hospitalization that can provoke anxiety. The first is the presence of pain. Interventions to reduce anxiety should also ensure that pain management is optimized [26]. Many papers examine interventions geared towards painful procedure related anxiety, most often in the emergency room or pre-operative settings [41,42]. Non-pharmacologic, psychosocial interventions are being offered more routinely for pre-procedure anxiety such as access to child life specialists, hospital clowns, parental presence, distraction with media, music, aromatherapy or coaching [43,44,45,46]. However, providing this type of psychosocial therapy in settings other than before a painful procedure is notably less common.

We discovered a single trial that offered child life specialists for anxiety not specifically related to a procedure, though it was in the emergency department, and they found a reduction in state anxiety when compared with hospital clowns and controls [11]. In light of our findings, we agree with their suggestion for more systematic assessment and identification of anxiety for pediatric patients and would add that this is needed for inpatient settings as well.

One of the limitations of our study is that non-English speaking patients were excluded. Another was proxy reporting. Although proxy measures were validated (currently under peer-review), it is possible that caregivers under- or over-estimated symptom severity, as this is a widely encountered issue in the literature [47]. In a recent validation study of the PAFS, no correlation was found between child and parent proxy measurement of anxiety and parents significantly over-estimated their child’s anxiety [17]. This may have affected symptom scores of younger patients, such as those in general pediatrics and cardiology, who rely on parent proxy reports of symptoms.

## 5. Conclusions

This study shows that PNVA are commonly experienced symptoms among pediatric inpatients and that anxiety is the most common and severe of these. Anxiety, in particular, needs to be more effectively anticipated, identified and treated. Effective and safe approaches to management approaches should consider integrating both pharmacological and non-pharmacological interventions. 

## Figures and Tables

**Table 1:** Patient characteristics, displayed by admission department.

Patient Demographics	*n* (%) or Mean (SD) or Median (IQR)			
	All Departments	Oncology	General Pediatrics	Cardiology
Patients	496	134	242	120
Age, years	5.1 (5.7)	8.6 (5.9)	4.2 (5.3)	2.6 (4.0)
Verbal	185 (37.3)	86 (64.2)	76 (31.4)	23 (19.2)
Gender				
Female	239 (48.2)	61 (45.5)	129 (53.3)	49 (40.8)
Ethnicity				
African	15 (3.0)	2 (1.5)	9 (3.7)	4 (3.3)
Arabic	3 (0.6)	0	3 (1.2)	0
Caucasian	287 (57.9)	68 (50.7)	144 (59.5)	75 (62.5)
Chinese	5 (1.0)	1 (0.7)	2 (0.8)	2 (1.7)
East Indian/Pakistani	19 (3.8)	2 (1.5)	11 (4.5)	6 (5.0)
Filipino	42 (8.5)	28 (20.9)	10 (4.1)	4 (3.3)
First Nations	43 (8.7)	4 (3.0)	31 (12.8)	8 (6.7)
Latin American/Mexican	7 (1.4)	2 (1.5)	1 (0.4)	4 (3.3)
Other	8 (1.6)	3 (2.2)	3 (1.2)	2 (1.7)
Identifies with multiple	67 (13.5)	24 (17.9)	28 (11.6)	15 (12.5)
First Admission to Hospital	266 (53.6)	19 (14.2)	178 (35.9)	69 (57.5)
Length of Stay, days	3 (2–7)	3 (2–6)	4 (3–8)	3 (2–9)

**Table 2:** Symptom prevalence and severity for all patients.

Symptom Measurements	*n* (%) or Mean (SD)		
	Pain (*n* = 496)	Nausea (*n* = 185)	Anxiety (*n* = 185)
Patients Symptomatic During Admission	256 (52.0)	102 (55.1)	125 (67.6)
Percentage of Days with Symptom	28.2 (35.8)	32.3 (37.8)	46.0 (37.4)
Symptom Severity on Symptomatic Days (/10)	3.0 (1.5)	3.2 (2.1)	3.7 (2.5)

**Table 3:** Symptom prevalence and severity.

Symptom	Oncology	General Pediatrics	Cardiology	*p*-Value
	Mean (SD) Percentage of Days Symptomatic			
Pain	22.7 (33.7)	30.7 (37.5)	26.1 (30.6)	0.098
Nausea	29.6 (34.0)	36.1 (39.5)	31.2 (32.9)	0.50
Anxiety	40.6 (41.6)	54.9 (39.3)	37.9 (43.2)	0.47
	**Mean (SD) Symptom Severity (/10)**			
Pain	2.7 (1.5)	3.4 (1.5)	3.3 (1.5)	0.008
Nausea	2.8 (2.6)	3.5 (1.6)	4.3 (1.7)	0.006
Anxiety	3.3 (2.3)	4.0 (1.8)	4.2 (4.4)	0.11

**Table 4:** Medication use for PNVA.

Symptom or Medication	Number (%) Patient Days			
	Oncology	General Pediatrics	Cardiology	*p*-Value
Patient Days	704	1451	1092	
Reported Pain	160 (22.7)	445 (30.7)	285 (26.1)	
Non-opioid Analgesics	69 (9.8)	414 (28.5)	473 (46.0)	<0.001
Opioid	127 (18.1)	83 (5.7)	262 (24.0)	<0.001
Reported Nausea	208 (29.6)	524 (36.1)	340 (31.2)	
Anti-emetics	373 (53.0)	51 (3.5)	370 (33.9)	<0.001
Reported Anxiety	286 (40.6)	797 (54.9)	414 (37.9)	
Anxiolytics/sedatives	37 (5.2)	52 (3.6)	182 (16.7)	<0.001
